# Psychometric properties of Multidimensional Health Locus of Control - A and General Self-Efficacy Scale in civil servants: ELSA-Brasil Musculoskeletal Study (ELSA-Brasil MSK)

**DOI:** 10.1590/bjpt-rbf.2014.0177

**Published:** 2016-06-30

**Authors:** Luciana A. C. Machado, Rosa W. Telles, Luciana Costa-Silva, Sandhi M. Barreto

**Affiliations:** 1 Departamento de Medicina Preventiva e Social, Faculdade de Medicina, Universidade Federal de Minas Gerais (UFMG), Belo Horizonte, MG, Brazil; 2 Departamento de Clinica Médica, Faculdade de Medicina, UFMG, Belo Horizonte, MG, Brazil; 3 Departamento de Propedêutica Complementar, Faculdade de Medicina, UFMG, Belo Horizonte, MG, Brazil

**Keywords:** psychometric properties, measurement instruments, health-related control beliefs, self-efficacy, ELSA-Brasil

## Abstract

**Background:**

Health-related control and self-efficacy beliefs can be assessed in the general population using Multidimensional Health Locus of Control-A subscales (MHLC-A) and the General Self-Efficacy Scale (GSES), respectively.

**Objective:**

To test construct validity, internal consistency, reliability (test-retest) and ceiling and floor effects of Portuguese-Brazil versions of MHLC-A and GSES.

**Method:**

Civil servants (*N*=2901) enrolled in a large Brazilian cohort were included. A new version of the GSES was produced (GSES-Brazil). Procedures for cross-cultural adaptation and testing of psychometric properties followed well-accepted international guidelines.

**Results:**

Confirmatory factor analyses yielded the following indices: MHLC-A (tridimensional model): χ^2^[*df*]=223.45[132], *p*-value <0.01; CFI=0.87; TLI=0.85; RMSEA=0.07 (0.07-0.08); WRMR=3.00. GSES-Brazil (unidimensional model): χ^2^[*df*]=788.60[35], *p*-value <0.01; CFI=0.95; TLI=0.94; RMSEA=0.09 (0.08-0.09); WRMR=2.50. Cronbach’s alpha coefficients and Intraclass Correlation Coefficients (ICC_2,1_) ranged from 0.57 (0.54-0.59) and 0.57 (0.47-0.65) for MHLC-A internality to 0.80 (0.79-0.81) and 0.71 (0.66-0.77) for GSES-Brazil, respectively. There was no evidence of ceiling and floor effects. Convergent validity analyses provided further support for construct validity of both scales.

**Conclusion:**

These findings support the use of the newly developed version of GSES-Brazil for the assessment of general self-efficacy of adult Brazilians. Internal consistency was lower than ideal for MHLC-A, indicating these subscales may need further refinements to provide a more psychometrically sound measure of control beliefs.

## BULLET POINTS

• Health-related control and self-efficacy beliefs can influence engagement.
• Psychometric properties of two scales designed to assess beliefs were tested.
• Civil servants enrolled in a large cohort (*N*=2901) were included.
• The GSES-Brazil showed acceptable properties for use in adult Brazilians.
• MHLC-A subscales need further refinements for the measure of control beliefs.

## Introduction

Engagement is one of the core elements of the chronic care model, which is the framework for patient-centered disease management in modern healthcare^1^ ^-^ ^3^. When it comes to the patients’ perspective, engagement can be understood under a tripartite definition, which includes their recognition of the importance to play an active role in their own health and health care, their self-management capability, and health-promoting behaviors^4^. Increasing attention has been given to the development of strategies that empower individuals to engage in the prevention and management of long-term illnesses^5^ ^,^ ^6^. This has also been recently set as a major goal for the physical therapy profession. According to the president of the World Confederation for Physical Therapy (WCPT), physical therapists are urged to discuss efficient ways “[...] to work with patients and clients to ensure that the health behavior changes that are important to sustainable healthy lives are *owned* by them”^7^.

Health locus of control and self-efficacy are psychological constructs derived from social learning theories^8^ that can influence engagement. Health locus of control refers to cognitions regarding the location where one’s control over health resides: in the individual or in external factors over which the individual has little control^9^. Bandura has described self-efficacy as the “[...] belief in one’s capabilities to organize and execute the courses of action required to manage prospective situations [...]” (p. 2)^10^. Individuals with high self-efficacy are regarded as those who believe in their competency to deal with life stressors^11^. Thus, health locus of control and self-efficacy relate to different phenomena that can interact to produce and to maintain changes in health behavior^12^ ^,^ ^13^. For example, a person with strong beliefs in his/her own actions to determine health outcomes (i.e. internal locus of control) and with a high sense of personal mastery over the environment and health (i.e. high self-efficacy) would be more likely to engage in the necessary behaviors to preserve or restore health. This has been supported by cumulative evidence from studies linking these two constructs to treatment adherence and long-lasting healthy behaviors^14^ ^-^ ^21^. However, the way health locus of control and self-efficacy interrelate and how they can affect both the initiation and persistence of certain behaviors (e.g. those that will ultimately influence health and treatment outcomes) are still not clearly understood. Limited evidence has pointed towards a mediation role of self-efficacy in providing control of one’s performance in face of stressful situations^22^.

Multidimensional Health Locus of Control scales (MHLC) have been developed for the assessment of health-related control beliefs in the general population (MHLC-A and MHLC-B)^23^ or in individuals with a pre-existing medical condition (MLHC-C)^24^. MHLC-A and MHLC-B are considered equivalent forms of the same scale, although the former has been more frequently used in the literature. MHLC scales measure the level of agreement with belief statements indicating internal or external health-control beliefs^23^. Self-efficacy can be assessed by the General Self-Efficacy Scale (GSES)^25^, which measures the generalized sense of self-efficacy according to whether individuals agree with statements reflecting a wide range of stressful situations, such as unexpected life events. Self-efficacy can also been assessed by context-specific scales, which measure the sense of personal competence to self-manage specific chronic health conditions (e.g. arthritis^26^, asthma^27^, diabetes^28^ ^,^ ^29^, hypertension^30^), to cope with persistent symptoms (e.g. pain^31^) and/or to perform certain behaviors (e.g. to exercise^32^ or return to work^33^).

Given that MHLC-A and GSES have been widely used to assess health beliefs and do not have their application restricted to a target population, these scales were among the instruments selected for use in an ancillary study on musculoskeletal disorders from a large cohort investigating risk and prognostic factors for chronic non-communicable diseases in Brazil: the ELSA-Brasil Musculoskeletal Study (ELSA-Brasil MSK)^34^ ^-^ ^36^. Both scales have been previously adapted for use in the Brazilian population and have had some of their psychometric properties tested (e.g. construct validity and internal consistency)^37^ ^-^ ^41^. However, the investigation of key properties of the scales was still lacking, such as test-retest reliability and ceiling and floor effects. Additionally, the presence of important semantic and scoring differences between the two available versions of the GSES^38^ ^,^ ^40^ raised questions on the adequacy of previous adaptation procedures.

The present study aimed to expand prior psychometric research on Portuguese-Brazil versions of MHLC-A and GSES by evaluating measurement properties not previously investigated in the literature, and to test construct (structural and convergent) validity and internal consistency of both scales in participants of ELSA-Brasil MSK. A new process of cross-cultural adaptation of the GSES was also performed prior to psychometric testing of this particular scale.

## Method

The study was approved by the ethics and research committee of Universidade Federal de Minas Gerais (UFMG), Belo Horizonte, MG, Brazil (protocol CAAE 0186.1.203.000-06). All participants signed a written informed consent after they had been informed of the nature and details of the study.

### Cross-cultural adaptation of GSES

Forward-translation and back-translation of the GSES were performed according to recommendations of the guideline for the process of cross-cultural adaptation of self-report measures^42^ (i.e. translators’ mother tongue was the target language and they were neither aware of nor informed about the concept of self-efficacy), except for the use of one bilingual translator in either translation process. The use of two translators was judged unnecessary given that previous Portuguese-Brazil versions of the scale^38^ ^,^ ^40^ could be used for comparison with the newly developed version. This was done by an expert committee formed by the translators and the authors of this study, in order to evaluate the semantic, idiomatic, experiential and conceptual equivalence between the original and all translated versions of the scale, thus allowing the production of the final version for psychometric testing.

The full versions of the scales previously adapted by Souza and Souza^38^ and Sbicigo et al.^40^ were retrieved through personal communication with the authors. Relevant differences between the two scales included the concept of effort of the original scale (e.g. “I can always manage to solve difficult problems *if I try hard enough*”), which was reflected in the version of Souza and Souza^38^ but not in the version of Sbicigo et al.^40^, and the inclusion of an additional response option (“neither agree nor disagree”) to the scoring of the scale adapted by Souza and Souza^38^.

The new version of the Portuguese-Brazil GSES (GSES-Brazil) was pretested on a socioeconomically heterogeneous sample of 15 adults recruited by convenience at the institution where the study was conducted. No relevant issues were identified in the comprehension of the newly developed version of the scale. The GSES-Brazil is described in Appendix 1.

### Psychometric testing of MHLC-A and GSES-Brazil

#### Participants and data collection

Active or retired civil servants enrolled in ELSA-Brasil at the Investigation Center of Minas Gerais (IC-MG), who returned for the second wave of face-to-face interviews and examination of the cohort between September 2012 and October 2014 (corresponding to baseline assessments of ELSA-Brasil MSK), were eligible for inclusion^34^ ^,^ ^35^. Data on health-related control and self-efficacy beliefs were collected by trained and certified staff through the application of the MHLC-A and GSES-Brazil during an initial interview at the subject’s workplace (initial test). For reliability analyses, a subsample of consecutive subjects who visited IC-MG for further assessments was requested to complete both scales a second time (retest). The time between the initial test and the retest could not be defined *a priori*, as it was dependent on the date scheduled for participants’ return to IC-MG to complete the battery of interviews and examination of the second wave of ELSA-Brasil^36^.

The targeted number of participants for test-retest reliability analyses was set at 300, representing three times the minimum sample size considered adequate by the COnsensus-based Standards for the selection of health Measurement INstruments (COSMIN) checklist^43^ ^,^ ^44^.

#### Instruments

The MHLC-A scale adapted to Portuguese-Brazil by Dela Coleta^37^ was used for the assessment of health-control beliefs. The MHLC-A includes 18 belief statements subdivided into three 6-item subscales: (1) internality, or the level of control attributed to the individual, e.g. “If I get sick, it is my own behavior that determines how soon I get well again”; (2) externality-powerful others, or the level of control attributed to other persons (e.g. health providers, social leaders), e.g. “Having regular contact with my physician is the best way for me to avoid illness”; and (3) externality-chance, or the level of control attributed to luck, fate or chance, e.g. “No matter what I do, I 'm likely to get sick”. Individuals should indicate whether they agree or disagree with each statement on a 6-point Likert scale (1 = strongly disagree; 2=moderately disagree; 3=slightly disagree; 4=slightly agree; 5=moderately agree; 6=strongly agree). The total MHLC-A score for each subscale ranges from 6 to 36 points, with higher scores indicating stronger beliefs in the control attributed to the particular dimension assessed by the subscale^45^.

The GSES-Brazil was used to assess general self-efficacy (Appendix 1). Similarly to the original scale^25^, the GSES-Brazil contains 10 statements on the self-perceived competence of the individual to deal with new or difficult tasks, e.g. “I can remain calm when facing difficulties because I can rely on my coping abilities”. The scoring structure of the original scale was also retained, as follows: 1=not at all true; 2=hardly true; 3=moderately true; 4=exactly true. The sum of the responses provides a total score ranging from 10 to 40 points, with higher scores indicating a stronger sense of self-efficacy.

#### Statistical analysis

The responses of the participants to the initial test were used for the analyses of construct (i.e. structural and convergent) validity, internal consistency and ceiling and floor effects. Participants who completed the same scale on two occasions (i.e.test and retest) were included in test-retest reliability analyses.

Structural validity was analyzed by confirmatory factor analyses (CFA). This approach has been recommended when the instrument to be tested has already had its factor structure determined by previous research^46^. The original three-factor and one-factor structures of MHLC-A and GSES-Brazil, respectively, were considered for CFA analyses. The diagonal weighted least squares estimation (DWLS) method with polychoric correlations was used to estimate the factorial model parameters. To evaluate the adequacy of the model, the following goodness to fit statistics were considered: χ^2^ (minimum fit function); comparative fit index (CFI); Tucker-Lewis index (TLI); root mean square error of approximation (RMSEA) with its associated 90% confidence interval (CI) and significance level due to a close fit test; weighted root mean square residual (WRMR). The χ^2^ statistic is an absolute measure of the discrepancy between the observed covariance matrix and the matrix the model predicts, with smaller χ^2^ values and higher probability levels representing better fit of the hypothesized model^47^ ^,^ ^48^. The CFI and TLI are incremental relative fit indices that estimate differences between the examined model and a hypothetical null model with unrelated components^47^. CFI and TLI values ≥0.95 are indicative of close fit^49^; however, less conservative cutoffs of 0.80 for TLI and 0.90 for CFI have also been used to indicate a desirable model fit^50^ ^,^ ^51^. The RMSEA considers a hypothetical null model where every component is related to other components in the model, and its estimate can be used both descriptively and inferentially^52^. RMSEA values ≤0.05 indicate a close fit, whereas values up to 0.08 indicate an adequate fit^53^. WRMR is a residual-based fit index for ordinal data that measure the (weighted) average discrepancy between the studied sample and the estimated population variances and covariances^48^. WRMR values close to 1.0 or lower denote adequate model fit^48^ ^,^ ^54^.

The Pearson correlation coefficient (*r*) was used to investigate convergent validity. To do this, the following a priori hypotheses were formulated: (1) the MHLC-A internality score correlates positively with the GSES-Brazil score (given that beliefs in internal control and self-efficacy are related but not identical constructs, the correlation between them were expected to be moderate; i.e. 0.3≤*r*≤0.6); (2) the magnitude of the correlation between the GSES-Brazil score and the MHLC-A internality score is higher (≥10%) than that between the GSES-Brazil score and the MHLC-A externality scores (for powerful others and chance subscales).

Internal consistency was analyzed by calculating Cronbach’s alpha coefficients and 95% CI, with coefficients between 0.70 and 0.95 considered satisfactory^46^. Ceiling and floor effects were analyzed according to the percentage of participants who achieved the lowest or highest possible scores on each (sub)scale, and were considered present if more than 15% of the sample scored the maximum or minimum points. Test-retest reliability was analyzed by type 2,1 Intraclass Correlation Coefficient (ICC_2,1_). Reliability was considered poor if ICC_2,1_<0.40, moderate if 0.40≤ICC_2,1_<0.75, substantial if 0.75≤ICC_2,1_≤0.90 and excellent if ICC_2,1_>0.90^55^. Alpha coefficients, floor-ceiling effects and ICC were calculated separately for each of the three MHLC-A subscales (i.e. internality; externality-powerful others; externality-chance).

The Statistical Package for the Social Sciences (SPSS Inc., Chicago, USA, version 19.0) was used for all analyses except CFA. The latter was performed by using the latent variable analysis package for structural equation modeling (lavaan^56^) in the R software for statistical computing (version 3.2.0)^57^.

## Results

A total of 2901 civil servants, who were enrolled in ELSA-Brasil and completed MHLC-A and/or GSES-Brazil during baseline assessments of ELSA-Brasil MSK, were included in this study. Of these, 307 and 308 provided data for MHLC-A and GSES-Brazil, respectively, at two occasions (i.e. test and retest). Sociodemographic characteristics (i.e. sex, age, race/ ethnicity, social class, work status and nature of occupation) of the included participants are described in Table 1. Social class (i.e. low; middle; high) was coded as a summary measure based on the current occupation held by the participant, the expected income based on the education level (i.e. average market value), and the observed income^58^. The nature of occupation was categorized into four groups (i.e.routine, manual; non-routine, manual; routine, non-manual; and, non-routine, non-manual) according to the definitions proposed by Autor et al.^59^. Because of the small amount of missing data, imputation procedures were not required^60^ (i.e., item-level missing rate was only 0.2%).

**Table 1:** Sociodemographic characteristics of study participants.

**Characteristics**	***N* (%)**	
	**Overall sample (*N*=2901)**	**Subsample** **a** **(*N*=308)**
Sex		
Female	1534 (52.9)	152 (49.4)
Male	1367 (47.1)	156 (50.6)
Age, years, mean ± SD	56.0±8.9	53.4±8.1
Race/ ethnicity		
White	1417 (48.8)	170 (55.2)
Black	368 (12.7)	34 (11.0)
Brown (‘Pardo’)	998 (34.4)	97 (31.5)
Asian	64 (2.2)	5 (1.6)
Indigenous	15 (0.5)	0
Missing	39 (1.4)	2 (0.6)
Social class		
Low	522 (18.0)	38 (12.3)
Middle	1184 (40.8)	124 (40.3)
High	1170 (40.3)	141 (45.8)
Missing	25 (0.9)	5 (1.6)
Work status		
Active	2308 (79.6)	291 (94.5)
Retired	593 (20.4)	17 (5.5)
Nature of occupation^b^		
Routine, manual	343 (11.8)	22 (7.1)
Non-routine, manual	23 (0.8)	0
Routine, non-manual	764 (26.3)	57 (18.5)
Non-routine, non-manual	1746 (60.2)	224 (72.7)
Missing	25 (0.9)	5 (1.6)

a Subsample included in analyses of test-retest reliability;

b Data provided from participants with active work status.

Results of CFA for MHLC-A and GSES-Brazil are described in Table 2. For MHLC-A, the TLI and RSMEA indices indicated an acceptable global fit for a tridimensional model. Items loading on internality were {1,6,8,12,13,17}, on externality-powerful others were {3,5,7,10,14,18}, and, on externality-chance were {2,4,9,11,15,16}. For GSES-Brazil, the ability of the unidimensional model to reproduce the input covariance matrix was supported by the CFI and TLI indices. The results of χ^2^ and WRMR statistics indicated a misfit of the proposed models for both scales (Table 2).

**Table 2:** Goodness of fit indices from confirmatory factor analyses (CFA) of GSES-Brazil and MHLC-A.

**Goodness of fit statistic**	**MHLC-A** **N=2889**	**GSES-Brazil** **N=2884**
χ^2^	2235.45, *df*=132*	788.60, *df*=35*
CFI	0.87	0.95
TLI	0.85	0.94
RMSEA (90% CI)	0.07 (0.07-0.08)*	0.09 (0.08-0.09)*
WRMR	3.01	2.50

GSES-Brazil: Portuguese-Brazil General Self-Efficacy Scale. MHLC-A: Multidimensional Health Locus of Control - Form A. χ^2^: minimum fit function. CFI: comparative fit index. TLI: Tucker Lewis index. RMSEA: root mean square error of approximation. CI: confidence interval. WRMR: weighted root mean square residual. Data refers to the robust (scaled) test statistic for one-factor GSES-Brazil and three-factor MHLC-A.

* p<0.001.

Results of convergent validity analyses showed significant correlations at a *p*=0.01 level (2-tailed) between MHLC-A internality and GSES-Brazil scores (*r*=0.27, *p*<0.01), MHLC-A powerful others and GSES-Brazil scores (*r*=0.14, *p*<0.01) and MHLC-A chance and GSES-Brazil scores (*r*=0.07, *p*<0.01). Results of internal consistency, ceiling and floor effects and test-retest reliability analyses for MHLOC-A subscales and GSES-Brazil are described in Table 3. The median sample time between the test and the retest of both scales was 26 days (interquartile range, IQR 15 to 42). *Post-hoc* sensitivity analyses for test-retest reliability was performed excluding participants whose time between the first and second application of the scales was within the highest quartile (i.e. >42 days) to explore whether this could influence the ICC_2,1_ values. These analyses included 237 participants and new ICC_2,1_ values (95% CI) were only marginally different from those found in the main analyses, as follows: 0.57 (0.47-0.66) for MHLC-A internality; 0.72 (0.65-0.78) for MHLC-A powerful others; 0.63 (0.54-0.70) for MHLC-A chance; 0.71 (0.64-0.77) for GSES-Brazil.

**Table 3:** Psychometric properties (i.e. internal consistency, test-retest reliability and floor-ceiling effects) of MHLC-A subscales and GSES-Brazil.

**Property**	**MHLC-A**			**GSES-Brazil**
	**Internality**	**Powerful others**	**Chance**	
Internal consistency (95% CI)	0.57 (0.54-0.59)	0.65 (0.63-0.67)	0.65 (0.63-0.67)	0.80 (0.79-0.81)
Test-retest reliability (95% CI)	0.57 (0.47-0.65)	0.70 (0.64-0.75)	0.66 (0.59-0.72)	0.71 (0.66-0.77)
Ceiling and floor effects	no	no	no	no
Minimum score, *N* (%)	1 (0.03)	5 (0.2)	106 (3.7)	2 (0.1)
Maximum score, *N* (%)	0 (0)	0 (0)	0 (0)	82 (2.8)

MHLC-A, Multidimensional Health Locus of Control - Form A. GSES-Brazil, Portuguese-Brazil General Self-Efficacy Scale.

## Discussion

This study has investigated the construct validity and internal consistency of two belief scales (MHLC-A and GSES-Brazil) in a large, ethnically and socioeconomically diverse sample of Brazilian civil servants. It has also provided novel data on test-retest reliability and floor-ceiling effects of these instruments.

Prior evidence on structural validity of MHLC-A in Brazil came from the study by Paine et al.^39^, who performed exploratory factor analysis (EFA) on a sample of 280 civil servants, university students and wives of military personal. In accordance to the original structure of MHLC-A, the authors found three factors with eigenvalues greater that 1.00 that accounted for 25% of the total variance^39^. Additionally, a series of principal component analyses (PCA) performed by Dela Coleta^37^ and reported in two unpublished studies seem to support the three-factor structure of MHLC-A; however, the lack of detailed information on these analyses preclude any formal conclusions (these have been briefly described in a book chapter^37^, p. 226-227). When it comes to the GSES-Brazil, one previous study conducted by Leme et al.^41^ performed CFA to assess the scale’s structural validity in a sample of 447 Brazilian adolescents and found goodness to fit indices that support its original one-factor structure (e.g. CFI=0.95; RMSEA=0.06).

CFA in our study has somewhat confirmed the tridimensional and unidimensional structures of MHLC-A and GSES-Brazil, respectively, given than three of the investigated indices (CFI, TLI and RMSEA) were close to the cutoffs recommended for acceptable model fit. Although the significant *p*-values for χ^2^ (<0.001) would imply a model misfit for both scales, this statistic is known to have important drawbacks when the sample size is large (e.g. *N*>1000). For example, in this situation it may reflect statistically significant but negligible differences between the sample and the model implied matrices^61^. The same concern has been raised by Yu for the WRMR statistic^48^ (p. 41, 42), which has also presented values indicating a poor fit in our sample.

Although we found that internal consistency of MHLC-A subscales was not satisfactory (i.e. cronbach’s alpha coefficients <0.70), it was within the range reported in previous studies conducted in other Brazilian samples, as follows: 0.54 to 0.67 for MHLC-A internality, 0.62 to 0.71 for MHLC-A powerful others and 0.51 to 0.78 for MHLC-A chance^37^ ^,^ ^39^. The GSES-Brazil presented adequate internal consistency (alpha coefficient >0.80), a result that was also similar to that of previous studies^38^ ^,^ ^40^ ^,^ ^41^. Results of correlation analyses were in line with our pre-set hypotheses, providing further support for the validity of both scales. As expected, the strength of one’s beliefs in his or her ability to perform the behaviors required to produce desired outcomes (i.e. high self-efficacy) correlated moderately with higher levels of confidence in the ability to exercise control (i.e. high internality beliefs).

To our knowledge, our study was the first to investigate the test-retest reliability and ceiling and floor effects of Portuguese-Brazil versions of MHLC-A subscales and GSES-Brazil. All (sub)scales presented moderate test-retest reliability, with the ICC_2,1_ value for GSES-Brazil nearly reaching the cutoff for substantial reliability. Additionally, there was no evidence of ceiling and floor effects for any of the investigated (sub)scales. Given that the present study was designed as a secondary investigation nested within assessments of a large cohort^36^, the date of the retest for reliability analyses could not be chosen *a priori*. The use of an arbitrary time between the test and retest was an important limitation in our study. For example, one could question whether personal beliefs assessed by MHLC-A and GSES-Brazil would remain stable over several weeks. However, similar results of sensitivity analyses excluding participants with longer periods between the two applications of the scales suggested no effect of time on our reliability results.

The assessment of health-related control and self-efficacy beliefs in epidemiologic research including non-clinical samples is extremely important for increasing the understanding of the mechanisms by which maladaptive beliefs influence the onset and progression of chronic conditions commonly managed by physical therapists. Our findings support the use of the newly developed version of the GSES-Brazil for the assessment of general self-efficacy in adult Brazilians. On the other hand, the psychometric properties of MHLC-A subscales, particularly internal consistency, were found to be lower than ideal, not reaching the quality standards recommended by current guidelines on health measurement instruments. Therefore, MHLC-A subscales may need further refinements to provide a more psychometrically sound measure of health-related control beliefs in epidemiologic studies.

## Appendix 1 Portuguese-Brazil General Self-Efficacy Scale (GSES-Brazil).

### Escala de Autoeficácia Geral - Brasil (EAEG-Brasil)

Eu vou ler afirmativas sobre algumas situações de vida. Gostaria que o(a) Sr(a) me respondesse utilizando essas opções de resposta: não é verdade; dificilmente é verdade; é mais ou menos verdade; é totalmente verdade.

01. Eu sempre consigo resolver os problemas difíceis se eu tentar bastante.

02. Se alguém for contra mim, eu posso encontrar os meios e as formas de alcançar o que eu quero.

03. Para mim, é fácil me agarrar aos meus objetivos e atingir as minhas metas.

04. Eu tenho confiança que sou capaz de lidar bem com acontecimentos inesperados.

05. Graças às minhas habilidades, eu sei como lidar com situações imprevistas.

06. Eu posso resolver a maioria dos problemas se eu fizer o esforço necessário.

07. Eu consigo me manter calmo(a) para enfrentar dificuldades porque confio nas minhas habilidades.

08. Diante de um problema, geralmente eu consigo encontrar diversas soluções.

09. Se eu tiver um problema, geralmente eu consigo pensar em uma solução.

10. Geralmente eu consigo lidar com qualquer dificuldade que aparece no meu caminho.

**Table:**

Não é verdade	1
Dificilmente é verdade	2
É mais ou menos verdade	3
É totalmente verdade	4

## References

[1] Wagner EH, Austin BT, Korff MV (1996). Organizing care for patients with chronic illness. Milbank Q.

[2] Bodenheimer T, Wagner EH, Grumbach K (2002). Improving primary care for patients with chronic illness. JAMA.

[3] Coleman K, Austin BT, Brach C, Wagner EH (2009). Evidence on the Chronic Care Model in the new millennium. Health Aff (Millwood).

[4] Simmons LA, Wolever RQ, Bechard EM, Snyderman R (2014). Patient engagement as a risk factor in personalized health care: a systematic review of the literature on chronic disease. Genome Med.

[5] Jordan JE, Briggs AM, Brand CA, Osborne RH (2008). Enhancing patient engagement in chronic disease self-management support initiatives in Australia: the need for an integrated approach. Med J Aust.

[6] Milani RV, Lavie CJ (2015). Health care 2020: reengineering health care delivery to combat chronic disease. Am J Med.

[7] World Physical Therapy Day – WCPT (2015). Resources on why physical therapy matters.

[8] Rosenstock IM, Strecher VJ, Becker MH (1988). Social learning theory and the Health Belief Model. Health Educ Q.

[9] Luszczynska A, Schwarzer R (2005). Multidimensional health locus of control: comments on the construct and its measurement. J Health Psychol.

[10] Bandura A, Bandura A (1995). Self-efficacy in changing societies. Exercise of personal and collective efficacy in changing societies.

[11] Schwarzer R, Bäßler J, Kwiatek P, Schröder K, Zhang J (1997). The assessment of optimistic self-beliefs: Comparison of the German, Spanish, and Chinese versions of the General Self-efficacy Scale. Appl Psychol.

[12] Cross MJ, March LM, Lapsley HM, Byrne E, Brooks PM (2006). Patient self-efficacy and health locus of control: relationships with health status and arthritis-related expenditure. Rheumatology (Oxford).

[13] Bandura A (1977). Self-efficacy: toward a unifying theory of behavioral change. Psychol Rev.

[14] De Las Cuevas C, Penate W, Sanz EJ (2014). The relationship of psychological reactance, health locus of control and sense of self-efficacy with adherence to treatment in psychiatric outpatients with depression. BMC Psychiatry.

[15] Slovinec D’Angelo ME, Pelletier LG, Reid RD, Huta V (2014). The roles of self-efficacy and motivation in the prediction of short- and long-term adherence to exercise among patients with coronary heart disease. Health Psychol.

[16] Weng LC, Dai YT, Huang HL, Chiang YJ (2010). Self-efficacy, self-care behaviours and quality of life of kidney transplant recipients. J Adv Nurs.

[17] Meland E, Maeland JG, Laerum E (1999). The importance of self-efficacy in cardiovascular risk factor change. Scand J Public Health.

[18] Christensen AJ, Howren MB, Hillis SL, Kaboli P, Carter BL, Cvengros JA (2010). Patient and physician beliefs about control over health: association of symmetrical beliefs with medication regimen adherence. J Gen Intern Med.

[19] Fuertes JN, Mislowack A, Bennett J, Paul L, Gilbert TC, Fontan G (2007). The physician-patient working alliance. Patient Educ Couns.

[20] Lee YJ, Shin SJ, Wang RH, Lin KD, Lee YL, Wang YH (2016). Pathways of empowerment perceptions, health literacy, self-efficacy, and self-care behaviors to glycemic control in patients with type 2 diabetes mellitus. Patient Educ Couns.

[21] Luszczynska A, Sarkar Y, Knoll N (2007). Received social support, self-efficacy, and finding benefits in disease as predictors of physical functioning and adherence to antiretroviral therapy. Patient Educ Couns.

[22] Litt MD (1988). Self-efficacy and perceived control: cognitive mediators of pain tolerance. J Pers Soc Psychol.

[23] Wallston KA, Wallston BS, DeVellis R (1978). Development of the Multidimensional Health Locus of Control (MHLC) Scales. Health Educ Monogr.

[24] Wallston KA, Stein MJ, Smith CA (1994). Form C of the MHLC scales: a condition-specific measure of locus of control. J Pers Assess.

[25] Schwarzer R, Jerusalem M (1995). Generalized Self-Efficacy scale.

[26] Brady TJ (2011). Measures of self-efficacy: Arthritis Self-Efficacy Scale (ASES), Arthritis Self-Efficacy Scale-8 Item (ASES-8), Children’s Arthritis Self-Efficacy Scale (CASE), Chronic Disease Self-Efficacy Scale (CDSES), Parent’s Arthritis Self-Efficacy Scale (PASE), and Rheumatoid Arthritis Self-Efficacy Scale (RASE). Arthritis Care Res (Hoboken).

[27] Tobin DL, Wigal JK, Winder JA, Holroyd KA, Creer TL (1987). The “Asthma Self-Efficacy Scale”. Ann Allergy.

[28] Grossman HY, Brink S, Hauser ST (1987). Self-efficacy in adolescent girls and boys with insulin-dependent diabetes mellitus. Diabetes Care.

[29] Bijl JV, Poelgeest-Eeltink AV, Shortridge-Baggett L (1999). The psychometric properties of the diabetes management self-efficacy scale for patients with type 2 diabetes mellitus. J Adv Nurs.

[30] Ogedegbe G, Mancuso CA, Allegrante JP, Charlson ME (2003). Development and evaluation of a medication adherence self-efficacy scale in hypertensive African-American patients. J Clin Epidemiol.

[31] Anderson KO, Dowds BN, Pelletz RE, Edwards WT, Peeters-Asdourian C (1995). Development and initial validation of a scale to measure self-efficacy beliefs in patients with chronic pain. Pain.

[32] Dzewaltowski D (1989). Toward a model of exercise motivation. J Sport Exerc Psychol.

[33] Shaw WS, Reme SE, Linton SJ, Huang YH, Pransky G (2011). 3rd place, PREMUS best paper competition: development of the return-to-work self-efficacy (RTWSE-19) questionnaire--psychometric properties and predictive validity. Scand J Work Environ Health.

[34] Machado LA, Telles RW, Costa-Silva L, Barreto SM (2016). Perfil da coorte ELSA-Brasil musculoesquelético. Braz J Rheumatol.

[35] Telles RW, Costa-Silva L, Machado LA, Barreto SM (2016). Investigating osteoarthritis in a subcohort of the Brazilian Longitudinal Study of Adult Health: The ELSA-Brasil Musculoskeletal Study (ELSA-Brasil MSK). Osteoarthr. cartil.

[36] Schmidt MI, Duncan BB, Mill JG, Lotufo PA, Chor D, Barreto SM (2015). Cohort Profile: Longitudinal Study of Adult Health (ELSA-Brasil).. Int J Epidemiol.

[37] Dela Coleta M, Dela Coleta MF (2004). Locus de controle e saúde. Modelos para pesquisa e modificação de comportamentos de saúde: teorias, estudos, instrumentos.

[38] Souza I, Souza M (2004). Validation of the general Self-Efficacy Scale. Cienc Rural.

[39] Paine P, Pasquali L, Paulo SES, Bianchi AL, Solha AC (1994). Psychometric properties of the Brazilian Health Locus of Control Scale. Psychol Rep.

[40] Sbicigo J, Teixeira M, Dias A, Dell’Aglio D (2012). Propriedades psicométricas da Escala de Autoeficácia Geral Percebida (EAGP). PSICO.

[41] Leme VB, Coimbra S, Gato J, Fontaine AM, Del Prette ZA (2013). Confirmatory factor analysis of the generalized self-efficacy scale in Brazil and Portugal. Span J Psychol.

[42] Beaton DE, Bombardier C, Guillemin F, Ferraz MB (2000). Guidelines for the process of cross-cultural adaptation of self-report measures. Spine.

[43] Terwee CB, Mokkink LB, Knol DL, Ostelo RW, Bouter LM, de Vet HC (2012). Rating the methodological quality in systematic reviews of studies on measurement properties: a scoring system for the COSMIN checklist. Qual Life Res.

[44] Mokkink LB, Prinsen CA, Bouter LM, Vet HC, Terwee CB (2016). The COnsensus-based Standards for the selection of health Measurement INstruments (COSMIN) and how to select an outcome measurement instrument. Braz J Phys Ther.

[45] Wallston KA (2007). Multidimensional Health Locus of Control (MHLC) scales.

[46] Terwee CB, Bot SD, de Boer MR, van der Windt DA, Knol DL, Dekker J (2007). Quality criteria were proposed for measurement properties of health status questionnaires. J Clin Epidemiol.

[47] Cook KF, Kallen MA, Amtmann D (2009). Having a fit: impact of number of items and distribution of data on traditional criteria for assessing IRT’s unidimensionality assumption. Qual Life Res.

[48] Yu CY (2002). Evaluating cutoff criteria of model fit indices for latent variable models with binary and continuous outcomes.

[49] Hu L, Bentler P (1999). Cut off criteria for fit indexes in covariance structure analysis: Conventional criteria versus new alternatives. Struct Equ Modeling.

[50] Chibnall JT, Tait RC (2005). Confirmatory factor analysis of the Pain Catastrophizing Scale in African American and Caucasian Workers’ Compensation claimants with low back injuries. Pain.

[51] Hooper D, Coughlan J, Mullen M (2008). Structural Equation Modelling: guidelines for determining model fit. Electron J Bus Res Methods.

[52] Kelley K, Lai K (2011). Accuracy in parameter estimation for the root mean square error of approximation: sample size planning for narrow confidence intervals. Multivariate Behav Res.

[53] Browne M, Cudeck R, Bollen KA, Long JS (1993). Alternative ways of assessing model fit. Testing structural equation models.

[54] Reeve BB, Hays RD, Bjorner JB, Cook KF, Crane PK, Teresi JA (2007). Psychometric evaluation and calibration of health-related quality of life item banks: plans for the Patient-Reported Outcomes Measurement Information System (PROMIS). Med Care.

[55] Fleiss J (1986). The design and analysis of clinical experiments.

[56] Rousseel Y (2012). lavaan: an R package for structural equation modeling. J Stat Softw.

[57] R Development Core Team R: A language and environment for statistical computing.

[58] Camelo LV, Giatti L, Chor D, Griep RH, Benseñor IM, Santos IS (2015). Associations of life course socioeconomic position and job stress with carotid intima-media thickness. The Brazilian Longitudinal Study of Adult Health (ELSA-Brasil). Soc Sci Med.

[59] Autor DH, Levy F, Murnane RJ (2003). The skill content of recent technological change: an empirical exploration. Q J Econ.

[60] Schafer JL (1999). Multiple imputation: a primer. Stat Methods Med Res.

[61] Brown TA (2015). Confirmatory factor analysis for applied research.

